# AI and organoid platforms for brain-targeted theranostics

**DOI:** 10.7150/thno.123243

**Published:** 2026-01-01

**Authors:** Rui Ye, Yupei Zhang, Wan Xu, Li Lai, Zhongwei Zhang, Yan Chen, Shugang Qin

**Affiliations:** 1 State Key Laboratory of Oral Diseases & National Center for Stomatology & National Clinical Research Center for Oral Diseases, West China Hospital of Stomatology, Sichuan University, Chengdu 610041, Sichuan, China; 2 Department of Experimental Research, Sichuan Clinical Research Center for Cancer, Sichuan Cancer Hospital & Institute, Sichuan Cancer Center, University of Electronic Science and Technology of China, Chengdu 610040, China.; 3 Department of Head and Neck Surgery, Sichuan Clinical Research Center for Cancer, Sichuan Cancer Hospital & Institute, Sichuan Cancer Center, University of Electronic Science and Technology of China, Chengdu 610040, China.; 4 Department of Pediatrics, Air Force Hospital of the Western Theater of the Chinese People's Liberation Army, Chengdu 500643, China.; 5 Department of Critical Care Medicine, Frontiers Science Center for Disease-related Molecular Network, State Key Laboratory of Biotherapy and Cancer Center, West China Hospital, Sichuan University, Chengdu 610041, China; 6 Department of Pharmacy, Sichuan Clinical Research Center for Cancer, Sichuan Cancer Hospital & Institute, Sichuan Cancer Center, University of Electronic Science and Technology of China, Chengdu 610040, China.

**Keywords:** Artificial intelligence (AI), Nanomedicine, Blood-brain barrier (BBB), Brain organoids, High-throughput screening (HTS)

## Abstract

Developing therapies for complex brain diseases faces significant challenges due to biological complexity and the stringent blood-brain barrier. While nanomedicine holds promise, traditional R&D paradigms suffer from inefficiency. This review introduces an intelligent theranostic paradigm that integrates high-fidelity brain organoid models, high-throughput screening (HTS/HCS), and Artificial Intelligence (AI). In this closed-loop workflow, organoid platforms serve a diagnostic role, generating predictive data on nanomedicine performance. AI then provides therapeutic guidance by processing this data to drive rational drug design, synthesis, and interaction prediction. This AI-driven convergence is poised to significantly accelerate the development of precisely targeted and individualized nanomedicines, offering new hope for breakthroughs in treating brain diseases.

## 1. Introduction

**Figure 1:**
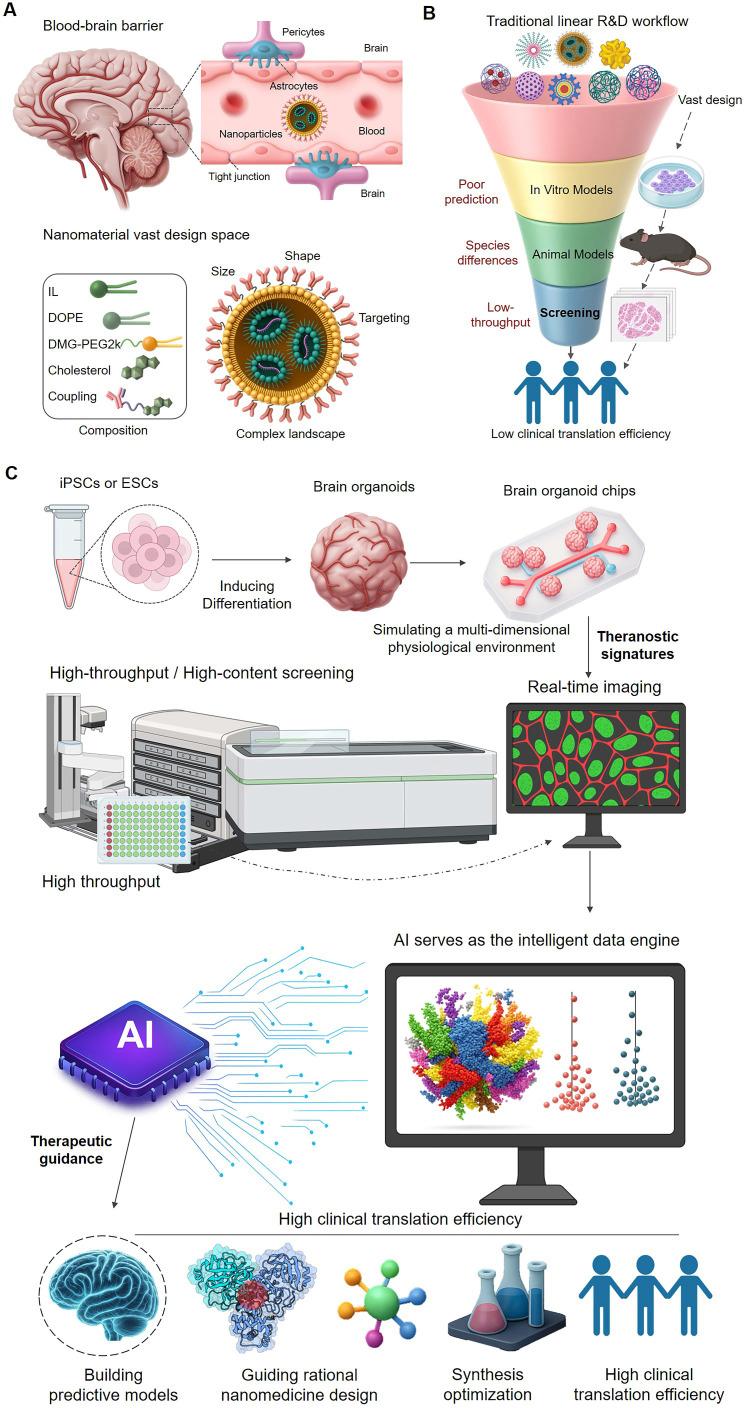
** From a linear workflow to an integrated theranostic paradigm.** (A) The Theranostic Challenge in Brain Delivery. The blood-brain barrier (BBB) presents a formidable obstacle. Compounding this challenge is the vast design space of nanoparticles (NPs), making it exceedingly difficult to predict which candidates will not only cross the BBB but also achieve the desired therapeutic effect. (B) The Disconnected Traditional R&D Workflow. The conventional linear process is inefficient because it separates testing from design. Its reliance on poorly predictive *in vitro* and animal models constitutes an unreliable 'diagnostic' step, leading to high failure rates and low clinical translation of potential 'therapeutics'. (C) The Integrated Theranostic Paradigm. This new approach closes the theranostic loop. High-fidelity models like brain organoids and organoid-chips serve as the 'diagnostic platform' to generate predictive data via high-throughput screening. Artificial Intelligence (AI) acts as the central engine, processing this diagnostic data to provide 'therapeutic guidance'—rationally designing and optimizing nanomedicines. This integration of diagnostics and therapeutics is poised to dramatically improve the success rate of developing effective brain-targeted nanomedicines.

Central Nervous System (CNS) diseases, encompassing a variety of complex conditions such as neurodegenerative diseases, brain tumors, and psychiatric disorders, represent a leading cause of long-term disability and mortality globally^1^, ^2^. Developing drugs for CNS conditions has historically been fraught with challenges due to the high complexity of the brain biological system, the heterogeneity of diverse cell types like neurons and glial cells, and the stringent restrictions imposed by the Blood-Brain Barrier (BBB)- a critical physiological barrier. The clinical trial failure rate for CNS drugs has been significantly higher than that for non-CNS drugs^3^. The BBB effectively prevents approximately 99% of small molecule drugs and nearly all large molecule biologicals from entering the brain, acting as the primary obstacle for drug delivery^4^, ^5^. Traditional drug discovery models have struggled to effectively overcome the BBB and precisely target therapeutic agents to diseased brain regions or cells^3^, leading to high clinical trial failure rates for CNS drugs. Furthermore, even when drugs reach the brain, non-specific distribution and targeting can result in severe off-target effects and adverse reactions, further narrowing the therapeutic window^6^. Therefore, developing novel therapeutic strategies capable of efficient brain delivery and precise targeting is crucial for conquering CNS diseases **(Figure 1A)**.

Nanomedicine, particularly engineered nanoparticle (NP) carriers, has shown immense potential^7^ for overcoming these challenges due to their tunable size, shape, surface properties, and capacity to conjugate diverse functional ligands^6^, ^8^. Nanoparticles can encapsulate various therapeutic agents (from small molecules to biological macromolecules), and by modifying their surface chemistry or conjugating targeting ligands, they can enhance their ability to cross the BBB, control drug release within the specific brain microenvironment, and potentially achieve precise targeted delivery to specific brain regions or cell types^9^ **(Figure 1A)**.

However, despite the great potential demonstrated by nanomedicine, traditional nanomedicine development paradigms still face inherent limitations that severely constrain their clinical translation efficiency and ultimate efficacy. These challenges span the entire pipeline from drug design and screening to application. Firstly, the complex interactions between nanoparticles and the BBB and its constituent cells (such as endothelial cells, pericytes, and astrocytes)^4^, as well as their transmembrane transport mechanisms^10^, ^11^, are difficult to accurately predict and control. Traditional design often relies on empirical optimization^12^ and lacks a deep understanding and efficient utilization of specific BBB transport mechanisms. Secondly, the complexity of nano-bio interactions poses another major challenge. Upon entering systemic circulation, NPs undergo a series of dynamic processes, including systemic clearance^5^, ^8^, ^12^, plasma protein corona formation^13^-^17^, and interactions with blood cells and endothelial cells^6^. These dynamic processes significantly influence NP biodistribution and targeting efficiency. Furthermore, the predictive power of existing preclinical models remains insufficient. Traditional *in vitro* cell models (such as 2D cell culture) struggle to mimic the complex microenvironment of the human brain^18^, ^19^ and BBB function^18^, ^20^, and animal models differ significantly from human brain structure and physiology, with critical divergences in aspects such as cortical folding (gyrification), the ratio of white to gray matter, and the diversity and function of glial cell subtypes^21^, ^22^. Consequently, nanomedicines showing promising results *in vitro* or in animal models often fail in clinical trials^3^. Additionally, the vastness of the nanomaterial design space necessitates exploring a massive number of parameter combinations, while the limited throughput of screening methods and the lack of intelligent tools to extract effective design rules from large datasets have made nanomedicine optimization processes slow and inefficient^23^. Finally, disease heterogeneity and inter-patient variability^24^ pose a formidable challenge to a "one-size-fits-all" R&D model **(Figure 1B)**.

Addressing the inherent limitations of traditional brain-targeted nanomedicine R&D concerning overcoming physiological barriers, predicting complex interactions, model predictive power, exploring the design space, and handling individual variability, incremental improvements based on single technologies are insufficient. The true "path to breaking the mold" lies in building a new R&D ecosystem that integrates multidisciplinary cutting-edge technologies and possesses intelligent decision-making capabilities. In recent years, the rapid development of high-fidelity *in vitro* models has provided an unprecedented platform for mimicking the real brain microenvironment and evaluating nanomedicine behavior *in vitro*. Combined with modern high-throughput screening (HTS) and high-content screening (HCS) technologies capable of generating massive high-content information, this has enabled the systematic generation of large-scale, multi-dimensional experimental data on the interactions between nanomedicines and complex brain biological systems. More critically, the rapid advancement of Artificial Intelligence (AI) has provided unprecedented opportunities for mining deep patterns from these massive, high-dimensional data, predicting nanomedicine properties and behavior, and guiding rational design and efficient screening, thereby becoming an intelligent engine for accelerating brain-targeted nanomedicine R&D. This integration heralds a paradigm shift towards an integrated, theranostic R&D workflow. In this new paradigm, the drug development process itself is reframed: the 'diagnostic' component involves rapidly and accurately predicting a candidate's biological behavior using high-fidelity models, while the 'therapeutic' component leverages these predictions to rationally design superior nanomedicines. This review will systematically discuss how these technologies can deeply collaborate, forming an integrated innovation paradigm for brain-targeted nanomedicine R&D** (Figure 1C)**.

## 2. Engineering human brain models for predictive theranostic insights

For decades, the study of the blood-brain barrier (BBB) and the effects of drugs on the central nervous system has relied on traditional preclinical models. These include two-dimensional (2D) *in vitro* systems, such as simple cell cultures or Transwell models^25^, and *in vivo* animal models. While foundational, these models have significant limitations. 2D cultures lack the complex 3D tissue architecture and cell-cell interactions of the human brain, leading to poor barrier function and physiologically irrelevant results. Animal models, though systemic, suffer from limitations such as high costs and low throughput^26^. More importantly, significant interspecies biological differences often lead to poor correlation with human outcomes. These differences are not trivial; for instance, the composition of blood plasma proteins varies between species, leading to the formation of a species-specific "protein corona" on nanoparticles^27^. This corona, in turn, dictates how the nanomedicine is recognized by the mononuclear phagocyte system (MPS), which also exhibits differences in cell populations and clearance activity^28^. Consequently, the aggressive off-target accumulation of nanomedicines in the liver and spleen observed in many small animal models is a frequent translational failure, as it may not accurately predict the biodistribution and therapeutic window in humans^29^.

To bridge this significant predictive gap, the development of more human-relevant and biologically complex models has become a critical priority^30^. In recent years, as a major breakthrough in three-dimensional (3D) cell culture technology, organoid technology has rapidly evolved, providing unprecedented theranostic platforms^31^-^34^. These "high-fidelity testing grounds" not only mimic complex physiological and pathological processes but also function as a 'diagnostic assay' for drug candidates, generating predictive data on their potential efficacy and toxicity. In the field of neuroscience, the progress of human brain organoids has been particularly noteworthy, and they have become indispensable new tools for clinical neuroscience research, bridging the gap between patient studies and animal models^35^ in** Table 1**.

### 2.1 Leveraging brain organoids for theranostic modeling of neuropathology

Brain organoids, as a 3D culture model capable of recapitulating the complexity of the human brain *in vitro*, have seen their construction methods evolve significantly, representing a major advancement in the field of neuroscience. This evolution has largely drawn upon and expanded the understanding of the self-organizing potential of pluripotent stem cells gained from earlier research. As reviewed by Eichmüller & Knoblich^35^, the initial landmark work can be traced back to the research by Sasai and colleagues, who pioneered the demonstration that mouse embryonic stem cells could self-organize under specific 3D culture conditions to form optic cups with stratified structures^36^ and cortical tissues with six layers^37^. hese early explorations established the methodological foundation for the subsequent construction of more complex brain organoids. Following this, the work by Lancaster *et al*. marked the formal establishment and widespread adoption of the "brain organoid" concept^38^, ^39^. They developed a method starting from human pluripotent stem cells to generate complex 3D structures containing multiple distinct brain regions, greatly advancing the *in vitro* modeling of early human brain development and related diseases. Building upon this, researchers have continuously optimized culture protocols, precisely controlling specific signaling pathways during early culture to guide stem cell differentiation towards specific brain regions (such as the cortex, hippocampus, and thalamus), forming brain organoids with greater regional specificity^40^-^53^. Concurrently, a method for constructing models directly from human fetal brain tissue also provided valuable supplementary resources for research. In recent years, Ramani *et al*. developed the "Hi-Q" brain organoid^54^, which, through further optimization of culture methods, achieved large-scale and reproducible generation of models with excellent cellular diversity and structural integrity, laying a solid foundation for subsequent high-fidelity disease modeling and drug screening.

The "high-fidelity" characteristics of brain organoids have been fully demonstrated in mimicking specific and complex physiological structures. Firstly, concerning the simulation of complex physiological barriers, brain organoids have shown unique advantages. The normal functioning of the brain relies heavily on the finely regulated protective structures like the blood-brain barrier (BBB) and the blood-cerebrospinal fluid barrier (B-CSF-B).

These barriers are not only critical for maintaining CNS homeostasis but also represent obstacles that many neurological disease treatments struggle to overcome. Pellegrini *et al*., in a pioneering study, successfully constructed human choroid plexus (ChP) organoids. These ChP organoids could not only form choroid plexus epithelium with a selectively permeable barrier but also secrete CSF-like fluid into the contained lumen^55^. Crucially, they found that the selective permeability of this *in vitro* constructed barrier to small molecules was highly consistent with the *in vivo* situation and could even predict the CNS permeability of novel compounds. This achievement clearly demonstrated the great potential of brain organoids (specifically, choroid plexus organoids in this context) in reconstituting complex, functional physiological barriers *in vitro*, providing valuable tools for studying barrier function, screening drugs capable of crossing the barrier, and understanding the role of barriers in disease.

Secondly, brain organoids have also demonstrated their exceptional "high-fidelity" in precisely mimicking the development and diseases of the nervous system, opening up new avenues for deeply understanding human-specific disease mechanisms. Eichmüller & Knoblich^35^, in their review, systematically summarized the successful applications of brain organoids in modeling various neurological diseases^35^. The scope broadly included viral encephalopathy^44^, ^56^-^62^, key pathological features of complex neurodegenerative diseases like Alzheimer's disease^63^-^68^, and various genetic neurodevelopmental syndromes caused by specific gene mutations^52^. Building on this, researchers have continuously pushed for methodological innovation to construct more refined and functional disease models. Notably, concerning malignant brain tumors like pediatric medulloblastoma and high-grade glioma, which traditional animal models struggle to fully recapitulate due to their human-specific complexity, Lago *et al*. pioneered the construction of specific brain region organoids from human iPSCs and combined this with gene editing technology to introduce pathogenic mutations, successfully establishing *in vitro* models of these pediatric brain tumors^69^. These highly relevant cancer organoids not only provide a platform for deeply studying the biological and genetic characteristics of tumors but also support various key downstream applications like *in vivo* transplantation, co-culture, lineage tracing, and drug screening, bringing new hope for conquering these challenging malignancies. Concurrently, to enhance the reproducibility and scalability of brain organoid models, Ramani *et al*. developed the "Hi-Q" brain organoid culture method^54^. By further optimizing culture medium components and methods, they achieved large-scale and reproducible generation of brain tissue models with rich cellular diversity and complex structure. These Hi-Q brain organoids not only successfully modeled key pathological features of neurodevelopmental defects like microcephaly but could also effectively recapitulate glioma invasion processes. This series of advancements further powerfully validated the core value of brain organoids in precisely mimicking physiological processes and the pathology of complex diseases, providing an unprecedented tool for neuroscience research and novel therapy development.

### 2.2 Limitations and improvement strategies for brain organoid

Although brain organoids have achieved remarkable success in mimicking tissue complexity, the technology is far from perfect, and acknowledging its current limitations is crucial for guiding future innovation. A primary challenge stems from their reliance on self-organization, which, while powerful, introduces significant cellular heterogeneity and batch-to-batch variability, complicating standardization and reproducibility.

Structurally, a major and widely recognized limitation of static organoid cultures is the lack of functional vascularization. As organoids grow beyond a few hundred micrometers, their internal regions suffer from insufficient nutrient and oxygen supply, leading to the formation of hypoxic or necrotic cores^70^. Concurrently, these *in vitro* culture conditions can induce significant cellular stress responses, further compromising the physiological relevance of the models^71^. Furthermore, current protocols often fail to generate the full spectrum of cell types found in the human brain; the insufficient integration of critical non-neuronal cells, such as microglia, oligodendrocytes, and pericytes, limits their ability to model complex cell-cell interactions, including neuroinflammation and myelination^55^, ^72^-^80^. Finally, most brain organoids recapitulate early fetal development and struggle to achieve a mature, adult-like state, which is a significant caveat for modeling late-onset neurodegenerative diseases. These collective challenges severely constrain the predictive power of standalone organoid models.

To overcome this critical bottleneck of avascularity, several innovative strategies have been actively pursued. A primary approach involves the co-culture of brain organoids with endothelial cells (e.g., human umbilical vein endothelial cells or iPSC-derived endothelial cells), often supplemented with supporting cells like mesenchymal stem cells or pericytes, to promote the self-assembly of capillary-like networks within the organoid tissue^81^-^83^. Concurrently, genetic engineering techniques have emerged as a powerful tool. By inducing the expression of key transcription factors, such as ETV2, pluripotent stem cells can be directly programmed towards an endothelial lineage, allowing for the in situ generation of vascular networks from the same cellular source as the neural tissue^84^.

### 2.3 Advancing theranostic platforms with brain-organoid-on-a-chip systems

**Figure 2:**
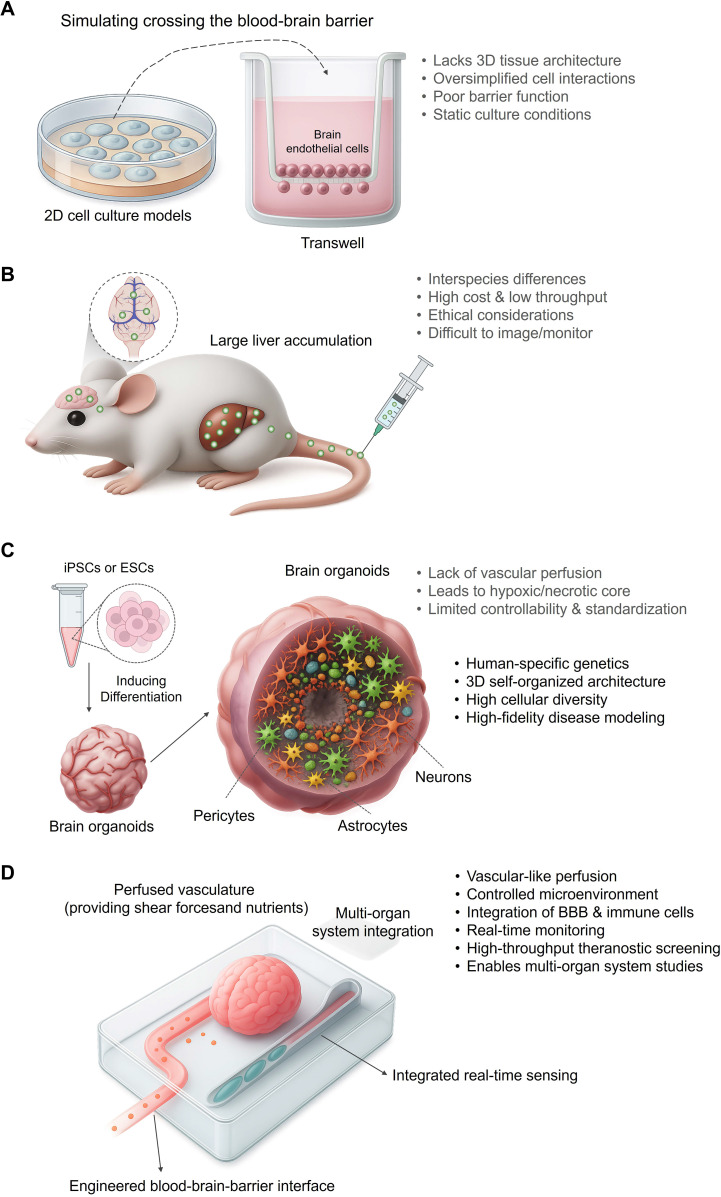
** The evolution of preclinical models toward high-fidelity theranostic platforms.** (A) Traditional *in vitro* models provide low-fidelity diagnostic data. Simple 2D cultures and Transwell systems, while foundational, suffer from a lack of 3D architecture, oversimplified cell interactions, and poor barrier function. These limitations severely reduce their predictive power for *in vivo* performance, yielding unreliable diagnostic information for candidate selection. (B) Animal models offer systemic context but with poor translatability. *In vivo* models present significant challenges, including interspecies differences, high costs, and ethical considerations. Crucially, they often fail to predict human-specific responses (e.g., nanoparticle accumulation in the liver instead of the brain), making them a poorly translated platform for developing human-targeted theranostics. (C) Brain organoids represent a leap toward human-relevant theranostic modeling. Derived from human iPSCs or ESCs, brain organoids recapitulate key features of human brain development, including human-specific genetics and complex cellular diversity. This enables high-fidelity disease modeling, offering a far more relevant context for evaluating nanomedicines. However, limitations such as a lack of vascular perfusion and limited standardization still hamper their full potential as robust theranostic platforms. (D) Brain-Organoid-on-a-Chip (BOoC) systems emerge as integrated theranostic platforms. By integrating organoids into microfluidic devices, BOoC technology overcomes many limitations of static cultures. It provides a controlled, perfused microenvironment, enables the integration of an engineered BBB, and allows for real-time monitoring. These features establish BOoC systems as the most advanced preclinical platforms for high-throughput, physiologically relevant screening of brain-targeted nanotheranostics.

While the aforementioned strategies show promise in forming structural vascular-like networks, achieving functional, dynamic perfusion that mimics physiological blood flow remains a significant hurdle^85^. To systematically overcome the bottlenecks this and other bottlenecks of traditional models** (Figure 2A, B)** and static organoid cultures **(Figure 2C)**, the "Brain Organoid-on-a-Chip (BOoC)" technology has emerged, representing the current state-of-the-art in preclinical brain modeling** (Figure 2)**^86^.

This innovative technology ingeniously combines the inherent biological complexity of organoids with the precision engineering advantages of organ-on-a-chip platforms. As highlighted by Vunjak-Novakovic *et al*. in their review on the progress of organ-on-a-chip^87^, organ-on-a-chip systems constructed using engineering methods like microfluidics can provide a more dynamic and controlled microenvironment for cell and tissue culture. Specifically for brain organoids, the BOoC platform can achieve fine-tuned control over their culture microenvironment, such as simulating physiological blood flow through perfusion systems, applying precise mechanical stimuli (e.g., shear stress), and easily integrating various sensors for real-time online monitoring of cell viability and key parameters^88^-^92^. For example, the practice by Cho *et al*. of integrating brain extracellular matrix with microfluidics to construct a blood-brain barrier model successfully promoted the structural and functional maturation of human brain organoids^93^. This demonstrated that this engineering method could effectively overcome challenges in traditional organoid culture related to imprecise environmental control, restricted nutrient transport, and difficulty in standardization, providing a powerful tool for constructing more functionally stable and physiologically relevant *in vitro* brain models (Table 2).

This strategy of integrating brain organoids with on-chip technology has shown great potential in addressing key bottlenecks in brain disease research and drug development, particularly in simulating the BBB and studying drug delivery. By co-culturing brain organoids with key cell types like brain endothelial cells, pericytes, and astrocytes on a chip, it is possible to build BBB models that are structurally more realistic and functionally more complete^94^. These BOoC-BBB models can not only highly mimic core physiological functions like BBB structural integrity and selective permeability but can also be used for dynamic studies of nanoparticle transcytosis mechanisms and efficiency. For instance, existing research constructed a multi-cellular co-culture system on a chip incorporating brain endothelial cells, pericytes, and a 3D astrocyte network, successfully recapitulating key structural and functional characteristics of the BBB, including tight barrier function, specific gene expression profiles, and physiologically relevant astrocyte polarization. Importantly, this platform could precisely track the 3D distribution of nanoparticles within the vascular and perivascular regions and reveal the mechanisms of cellular uptake and BBB penetration mediated by receptor-mediated transcytosis^94^. This undoubtedly provides a crucial *in vitro* evaluation tool for developing novel nanomedicines capable of effectively crossing physiological barriers.

Besides simulating the BBB and specific neurological diseases, a key development direction for BOoC technology is the construction of multi-organ interconnected systems comprising modules of multiple organoids or different tissue types, thereby more comprehensively mimicking complex physiological and pathological processes of the human body *in vitro*. The behavior of nanomedicines in the body often involves complex communication and interactions among multiple organs, which is difficult to fully capture with single-organ models^95^. By integrating key organ modules such as brain, liver, and kidney on a chip, researchers can more realistically simulate drug Absorption, Distribution, Metabolism, and Excretion (ADMET) processes and evaluate their efficacy and potential systemic toxicity in different organs^96^-^98^. These multi-organ interconnected BOoC systems provide an unprecedented platform, offering greater physiological relevance, for deeply understanding the overall behavior of nanomedicines in complex biological systems, revealing the interaction patterns between them and different cell types and distal organ microenvironments—that is, effectively addressing the complexity and unpredictability of nano-bio interactions^99^-^101^.

However, whether it is increasingly complex brain organoid models or precisely controlled brain organoid-on-a-chip systems, despite their immense potential in mimicking the real brain microenvironment and generating massive, high-dimensional, and dynamic biological data, challenges remained in efficiently leveraging this potential for large-scale drug screening and translating it into practical R&D breakthroughs. This collectively highlighted an urgent need: these high-fidelity *in vitro* models must be deeply integrated with high-throughput screening technologies and intelligent analysis platforms driven by artificial intelligence.

## 3. Powering the theranostic workflow with high-throughput screening platforms

**Figure 3:**
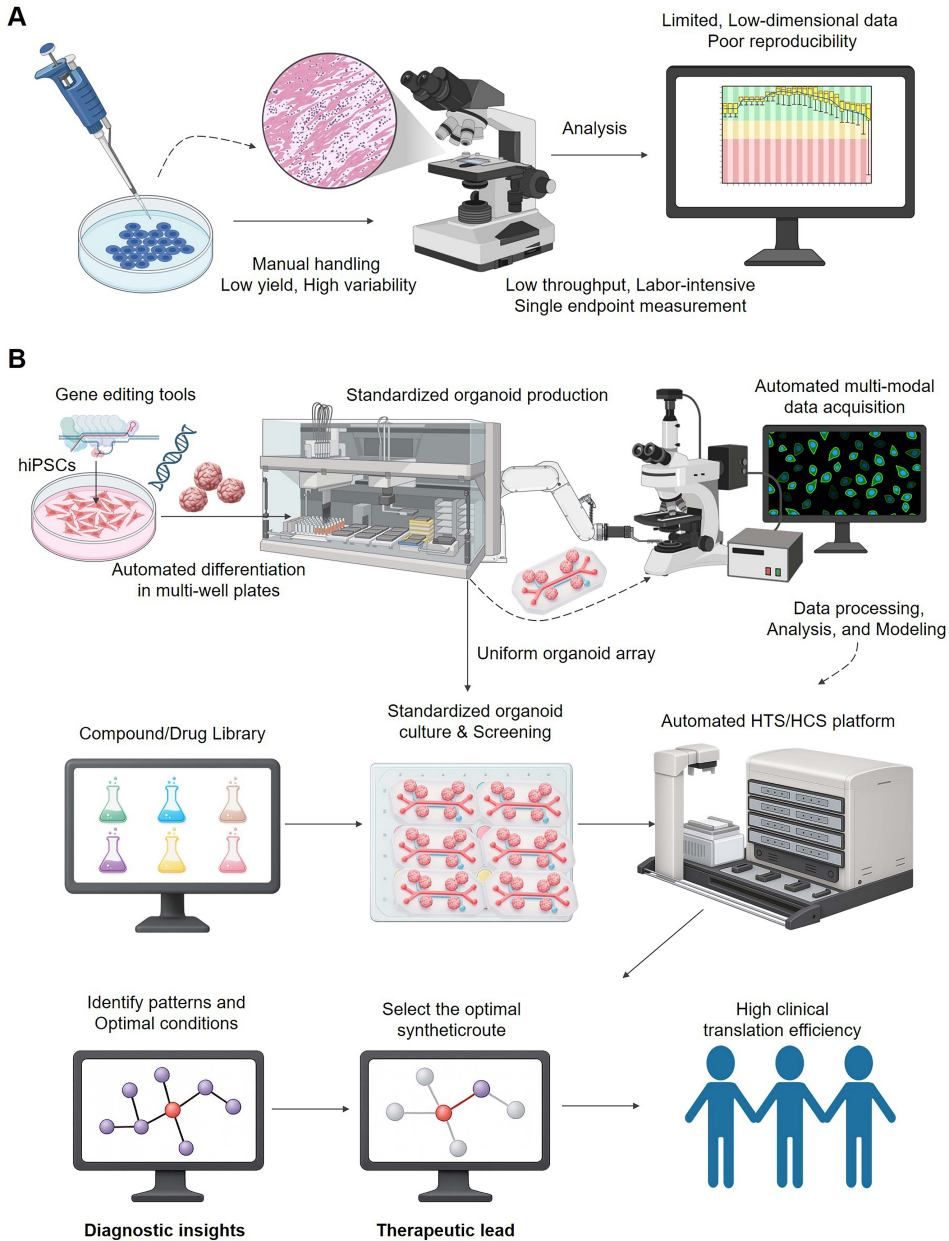
** From manual screening to an automated theranostic platform.** (A) The Traditional Manual Screening Workflow is inadequate for theranostic R&D. This process is characterized by manual handling, leading to low yield, high variability, and poor reproducibility. The reliance on labor-intensive, low-throughput analysis provides only limited, low-dimensional data, making it impossible to systematically evaluate the complex interactions required for developing effective theranostics. (B) The Automated HTS/HCS Platform Powers a Modern Theranostic Workflow. This integrated paradigm begins with the standardized, automated production of uniform organoid arrays, often from gene-edited hiPSCs. An automated HTS/HCS platform then screens large compound libraries against these cultures. The automated, multi-modal data acquisition generates large-scale, high-dimensional datasets, which serve as the 'diagnostic' input for analysis and modeling. This allows for the identification of biological patterns and the selection of optimal candidates, directly connecting the diagnostic screening to therapeutic development and significantly improving clinical translation efficiency.

To fully leverage brain organoids in drug development, a paradigm shift is required from traditional, manual, and low-throughput methods to integrated, automated platforms driven by High-Throughput Screening (HTS) and High-Content Screening (HCS) **(Figure 3)**. The conventional approach **(Figure 3A)** is often plagued by high variability, labor-intensive workflows, and limited data dimensionality, which severely constrains its scalability and predictive power. In contrast, the new paradigm **(Figure 3B)** integrates automation for standardized organoid production and screening with advanced, multi-modal data acquisition systems, paving the way for robust data analysis and, ultimately, higher clinical translation efficiency.

As discussed in the preceding chapter, while high-fidelity 3D cell models like brain organoids offered unprecedented physiological relevance compared to traditional methods, their application in drug development was hindered by several limitations when attempting large-scale screening. These prominently included the low throughput of existing culture and analysis methods, which made systematic large-scale drug screening infeasible; inherent challenges of the models themselves, such as cellular composition heterogeneity, size and morphological variability, lack of vascularization, and complex handling procedures^102^; and the difficulty in efficiently processing and extracting valuable information from the high-dimensional data they generated. These limitations severely constrained the application efficiency and data output of brain organoids in drug development. To overcome these challenges and accelerate the application of organoid models, the introduction of High-Throughput Screening (HTS) and High-Content Screening (HCS) technologies was essential. HTS, through its automation, miniaturization, and standardized workflows, significantly increased experimental volume, primarily breaking the throughput bottleneck in brain organoid research. HCS, on the other hand, further enabled the acquisition of rich, multi-dimensional phenotypic and mechanistic data from complex models. The integration of HTS and HCS thus brought unprecedented opportunities for understanding complex brain function and tackling neurological diseases by creating a powerful platform to leverage the potential of organoid models.

### 3.1 Scaling up theranostic discovery with high-throughput screening

The core capability of HTS is primarily reflected in its significant contribution to scaling up brain organoid research. By integrating automated culture systems, advanced microfluidic technology, and innovative methods like brain organoid-on-a-chip, researchers can now achieve large-scale generation, standardized maintenance, and precise manipulation of brain organoids, greatly overcoming the throughput bottleneck of traditional methods.

A prominent example is the "Hi-Q" brain organoid culture method developed by Ramani *et al*., which can efficiently and reproducibly generate thousands of brain organoids and has been successfully applied to high-throughput drug screening^54^, providing a solid methodological foundation for large-scale evaluation of nanomedicine behavior in complex 3D brain models (such as BBB permeability simulation, targeting efficiency, efficacy, and toxicity). This fully demonstrated how HTS technology, through standardized culture and handling procedures, overcomes the bottleneck of scalability in traditional brain organoid research. Additionally, Renner *et al*. reported a fully automated high-throughput workflow for chemical screening using human midbrain organoids^103^, which achieved complete automation from organoid generation and maintenance to optical analysis in standard 96-well plates, significantly improving experimental efficiency and reproducibility. This fully demonstrated how HTS technology, through standardized culture and handling procedures, overcomes the bottleneck of scalability in traditional brain organoid research **(Table 3)**.

### 3.2 Gaining deep theranostic insights with high-content screening

However, achieving scalability alone is insufficient; extracting valuable insights from large sample sets requires deep data. The complexity of 3D cell models necessitates HCS to capture rich, multi-parameter data, going beyond simple endpoint indicators^102^. In this regard, HCS plays a central role on the HTS platform. It transforms complex biological processes into precisely quantifiable and deeply analyzable data, achieving deep data generation for brain organoid research. HCS can rapidly and automatically acquire and quantify complex biological data from large numbers of brain organoid samples, including fine details of cellular morphology, localization of specific molecular markers, and cellular network states, far surpassing traditional single biochemical or viability metrics. Collectively, this rich, multi-parameter dataset constitutes a 'theranostic signature' of the nanoparticle's interaction with the biological system, providing the crucial information needed for subsequent AI-driven prediction.

Combining HCS with other functional high-throughput analysis methods enables a more comprehensive characterization of the brain organoid's biological state. In the automated workflow by Renner *et al*., multiple high-throughput analysis methods were integrated, including High-Content Imaging (HCI) for evaluating brain organoid cellular composition and neurite morphology, multi-electrode array (MEA) technology for measuring electrophysiological activity, and calcium imaging for single-cell neuronal activity analysis^103^. This multi-modal HTS integration method captured complex information from morphological, functional, and other levels in brain organoids, greatly improving the efficiency and depth of quantitative analysis of brain organoid phenotypes. The study also emphasized that by optimizing tissue clearing protocols and using thin-section brain organoid (SFEBs) models, they overcame the challenge of imaging thick tissues, achieving high-content imaging with single-cell resolution across the entire brain organoid, simplifying cumbersome steps like traditional sectioning and further improving throughput. Durens *et al*. similarly developed an integrated high-throughput workflow that applied HCI, MEA, and calcium imaging to hiPSC-derived brain organoids, revealing information on neuronal activity and cellular composition through multimodal analysis and further demonstrating the power of high-throughput methods in brain organoid research^104^.

Particularly for complex neurological diseases, HCS can quantify disease-related complex phenotypes or pathological features, making them directly applicable to drug screening and evaluation. For instance, Park *et al*. constructed a HCS platform based on human iPSC-derived brain organoids, utilizing 1300 brain organoids from 11 participants (including CRISPR-Cas9 edited isogenic lines) for large-scale testing of FDA-approved blood-brain barrier permeable drugs^105^. Using HCS, they successfully quantified Alzheimer's disease (AD)-related pathological features, such as Aβ and p-tau protein deposition and cell viability. This study not only demonstrated the feasibility and efficiency of quantitatively evaluating complex disease phenotypes using HCS on large numbers of brain organoid samples but also combined mathematical modeling and network analysis to guide drug selection, providing strong support for drug screening and precision medicine strategies based on brain organoids. This ability to convert biological phenomena into structured data not only enhances the objectivity and reproducibility of research but, more importantly, provides a solid data foundation for subsequent complex computational analysis, AI modeling, and intelligent decision-making.

### 3.3 Reaching molecular resolution in theranostic profiling with single-cell omics

At a deeper molecular level, modern HCS platforms are further integrating high-throughput omics technologies, particularly single-cell omics, to reveal molecular-level biological responses and mechanisms at single-cell resolution. This integration significantly enhances the depth and resolution of information obtained from brain organoid models, enabling an unprecedented fine-grained understanding of drug effects on cellular subtypes and molecular responses of specific cell types. These methods, applied to models like brain organoids, are helping us understand drug action at the cellular and molecular mechanisms with unprecedented detail. This powerful potential for deep mechanistic investigation provides unprecedented cellular and molecular level insights for brain-targeted drug development, directly guiding more precise and effective drug design and screening. For example, a recent study utilized CRISPR-Cas9 for high-throughput genetic perturbation of various brain disease-related genes in human brain organoids and, through single-cell RNA sequencing, deeply investigated the impact of gene functional loss on cell fate determination, cellular state, and gene regulatory networks^106^. This work demonstrated the capability to acquire and analyze complex single-cell molecular profiles in brain organoid HTS, revealing disease mechanisms and the vulnerability of cellular subtypes with unprecedented depth, providing new avenues for drug target identification and validation. Another study obtained dense time-point single-cell transcriptome and chromatin accessibility data during human brain organoid development and constructed gene regulatory networks to decipher molecular mechanisms of human brain development^107^. By combining high-throughput genetic perturbation with single-cell readout, they validated key transcription factors in cell fate determination. These two studies collectively emphasized the strong potential of utilizing HTS strategies to acquire high-dimensional single-cell molecular data from brain organoids, a physiologically relevant model, and combining this with computational methods for deep mechanistic investigation.

Consequently, the integration of HTS (providing scalability and automation) and HCS (providing high-content data and multi-modal analysis) constitutes a data platform capable of generating massive, high-quality, high-dimensional biological data, addressing the bottleneck in data analysis for 3D model HCS. This data, especially single-cell omics and network data, is key for training powerful AI algorithms. It can help us uncover complex interaction patterns between "nanomedicines and brain organoids," build precise predictive models, and ultimately guide rational nanomedicine design and virtual screening, further enhancing the intelligence level of drug development. However, to truly transform this powerful platform into a mature drug development tool, further in-depth and systematic investigation is needed concerning the correlation between the key *in vitro* phenotypes generated and clinical biomarkers, as well as the predictive applicability of high-throughput screening results for clinical efficacy^108^.

### 4 Orchestrating the theranostic paradigm with artificial intelligence

**Figure 4:**
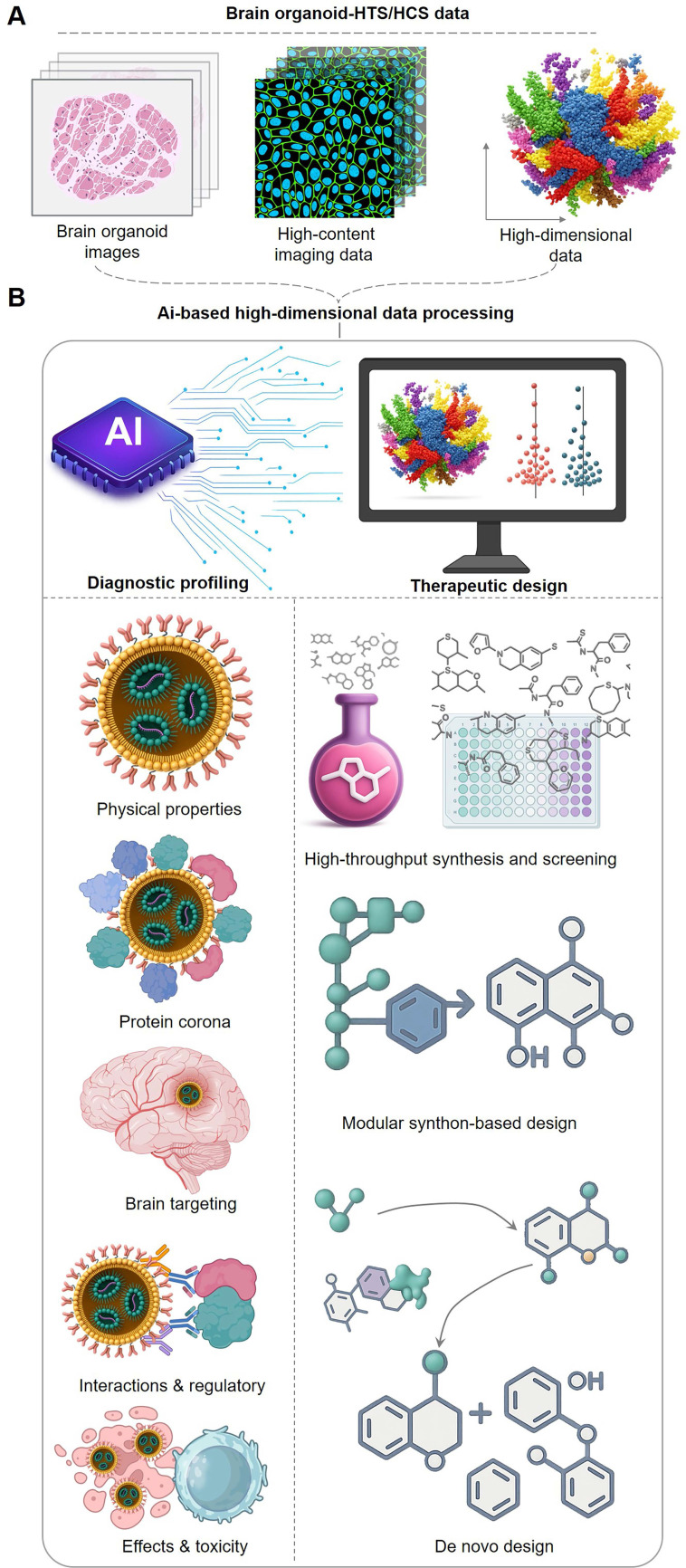
** The AI engine orchestrating the theranostic workflow.** The theranostic workflow is fueled by high-dimensional data generated from brain organoid-HTS/HCS platforms. This data, serving as the diagnostic input, is processed by an AI engine that performs two synergistic functions: Theranostic Prediction: AI models are trained to predict the complex *in vivo* behavior of nanomedicines. This includes forecasting their physical properties, protein corona formation, brain targeting efficiency, interactions with regulatory systems (e.g., immune cells), and overall efficacy and toxicity. These predictions form a comprehensive diagnostic profile for each candidate. Therapeutic Guidance: Based on the diagnostic insights, AI provides rational guidance for creating new and improved nanotherapeutics. This can involve high-throughput virtual screening, modular synthon-based design, or de novo design of novel molecular structures. This AI-guided cycle, which continuously refines therapeutic design based on diagnostic prediction, accelerates the discovery of effective and safe brain-targeted therapies.

The convergence of high-fidelity biological models with advanced computational methods gives rise to a powerful new R&D engine driven by Artificial Intelligence (AI). In this paradigm, complex, high-dimensional data generated from brain organoid-HTS/HCS platforms—including histological images, high-content imaging data, and multi-omics readouts—are fed into AI systems for processing and analysis. The AI then serves a dual purpose: it builds models to predict the complex biological behavior of nanomedicines and provides rational guidance for the de novo design and optimized synthesis of new therapeutic candidates, creating a powerful loop between data generation and intelligent design **(Figure 4)**. Building upon the robust data platform enabled by high-fidelity organoid models and HTS/HCS, AI takes center stage as the intelligent engine driving the design and screening of brain-targeted nanomedicines. AI, particularly through data-driven ML/DL methods, offers unprecedented capabilities for developing predictive models that accurately forecast the complex behavior and properties of nanomedicines within the brain. This includes predicting critical parameters such as BBB permeability, brain distribution, cell targeting, binding affinity to specific targets, and essential ADMET/PK characteristics. This powerful predictive capacity fundamentally transforms the R&D workflow, enabling a more intelligent and rational approach that directly guides the optimization of nanomedicine structure and composition towards desired brain targeting **(Table 4)**.

### 4.1 Extracting theranostic signatures from complex data using AI

The first crucial function of AI in this integrated paradigm is the efficient processing and extraction of meaningful insights from the massive, high-quality, high-dimensional biological data generated by the brain organoid and HTS/HCS platforms. This complex, human-relevant dataset is indispensable for training powerful AI algorithms to recognize biologically relevant patterns that would be imperceptible to human analysis.

For example, Convolutional Neural Networks (CNNs) are adept at processing high-content imaging data and can automatically identify subtle changes in cell morphology, subcellular structures, and even cellular network activity patterns^109^-^111^. Lampart *et al*., in their review, mentioned the use of CNNs for processing and analyzing brain organoid image data from HCS platforms^112^, and Gritti *et al*. developed MOrgAna^113^, a tool that uses machine learning for automatic segmentation and quantification of brain organoid images. AI has also been used to interpret and understand more complex biological response data. For instance, the study by Tebon *et al*. used machine learning-based image segmentation and classification algorithms to successfully achieve label-free, longitudinal quantitative monitoring of the biological mass changes of thousands of brain organoids, thereby revealing the heterogeneity of treatment resistance at single-organoid resolution^114^.

Beyond image data, AI also excels at processing other high-dimensional data. For example, multi-layer perceptron models can efficiently process large-scale fluorescence data generated by high-throughput droplet screening, quantifying the impact of different chemical compositions on complex biological reaction systems^115^. When dealing with complex single-cell data, Ramos Zapatero *et al*. used the dendrogram analysis method Trellis for analyzing complex single-cell data, achieving fine-grained analysis of drug effects on post-translational modification (PTM) signals, DNA damage, cell cycle, and apoptosis across multiple dimensions from a high-throughput mass cytometry platform^116^. These AI algorithms can automatically learn and extract key biological features and patterns from these diverse types of high-throughput, high-content data, laying a solid foundation for subsequent predictive model building. In this phase, the core role of AI is to efficiently and accurately extract key biological features and patterns from high-throughput experimental data, transforming biological phenomena into precisely quantifiable and deeply analyzable data^102^, setting the stage for subsequent intelligent prediction and design.

### 4.2 Guiding therapeutic design with AI-driven theranostic prediction

Leveraging the rich features extracted from the high-fidelity brain organoid models via HTS and HCS, data-driven AI/DL methods unlock significant potential in brain-targeted nanomedicine development^117^. Their core strength lies in building predictive models that accurately forecast nanomedicine behavior and properties within the complex human brain environment, encompassing key factors like BBB permeability, distribution, targeting, binding affinity, and ADMET/PK profiles^118^. This powerful predictive capacity is reshaping the R&D landscape, enabling a more intelligent and rational approach that directly guides the design and optimization of nanomedicines for effective brain targeting.

#### 4.2.1 Designing the molecular architecture of nanotheranostics

AI's predictive capability and insights are reflected in various specific strategies for guiding nanomedicine design and screening. Firstly, AI can be used to predict the key formation and physical properties of nanostructures and guide component selection and design accordingly. For example, when designing nanomedicines formed by component self-assembly, the molecular structure of precursor molecules (such as the drug itself) plays a key role in their assembly behavior and the final nanoparticle size, but this is difficult to predict. In this regard, Shamay *et al*. developed the Quantitative Structure-Nanoparticle Assembly Prediction (QSNAP) model, which accurately predicted assembly behavior and size based on molecular descriptors of drug molecules and guided drug payload selection, successfully applied to the design of targeted nanomedicines^119^.

Secondly, structure-based virtual ligand screening (VLS) is a mature method using computational prediction to guide drug design^117^. Its principle involves computationally docking potential ligand molecules into the 3D structure of target proteins to predict binding modes and strength, thereby enabling the rapid identification of potential active molecules from vast chemical libraries. With the accessible chemical space expanding to billions or even trillions of molecules^23^, efficient VLS strategies, increasingly leveraging AI and machine learning (ML) techniques, have become crucial for accelerating lead compound discovery. AI/ML enhances VLS by improving docking accuracy, filtering out less promising candidates early, and navigating ultra-large chemical spaces more effectively than traditional brute-force simulation. For example, there was research using ultra-large-scale virtual screening to successfully discover active molecules with novel chemical scaffolds from a library of hundreds of millions of compounds^120^.

Furthermore, AI is also driving more principle-based rational design methods. Unlike virtual screening which searches existing chemical libraries, these methods focus on *de novo* design or building molecules from smaller units based on fundamental principles. One such approach is Modular Synthon-Based Design, which involves using a predefined set of molecular fragments or "synthons" as building blocks to construct novel molecular structures. This method allows for the systematic exploration of a vast *synthesizable* chemical space by combining and optimizing these fragments. Modular Synthon-Based Design, such as exemplified by the V-SYNTHES methods^121^, can efficiently generate lead compounds with high activity and diversity and avoid costly custom synthesis. AI significantly empowers Modular Synthon-Based Design by assisting in the selection and design of optimal synthons, guiding the efficient combinatorial assembly of fragments, predicting the properties and synthesizability of generated molecules, and navigating the vast combinatorial space. This type of AI-guided method provides important guidance for the rational design of components (such as active molecules and functional ligands) for brain-targeted nanomedicines, enabling the construction of NPs with tailored functionalities and improved properties.

#### 4.2.2 Predicting the biological fate and efficacy of nanotheranostics

AI's predictive capabilities are crucial for understanding the complex biological interactions of nanomedicines at multiple levels, from molecules to cells. Accurately evaluating these interactions is vital for understanding how nanoparticles behave upon entering the body and how they affect specific biological components, which in turn influences their efficacy and potential off-target risks.

At the molecular level, AI aids in predicting the interactions between nanomedicines (or their components) and biomolecules. A key focus here is the prediction of protein corona formation upon systemic administration. ML models are applied to predict the composition and characteristics of the protein corona formed around nanoparticles, crucial for understanding their subsequent biological identity and fate^122^, ^123^. Leveraging datasets from advanced techniques like mass spectrometry-based proteomics, ML models show promise in enabling efficient and reliable prediction of protein adsorption onto nanoparticles and their associated impacts^124^, ^125^. Furthermore, AI is instrumental in accurately evaluating the binding interactions between nanomedicine components (such as drugs or targeting ligands) and specific biological target proteins. AI, especially deep learning models, can predict these binding events based on molecular and protein structures^126^-^128^. However, these models face challenges in generalizing to predict the binding behavior of novel molecules or proteins, sometimes learning "shortcuts" from training data. Advanced methods like AI-Bind are being developed to improve generalization and provide more reliable tools for precise design and safety assessment^129^.

Moving to the cellular level, AI is applied to predict various nano-cellular interactions, encompassing processes such as cell recognition, adhesion, and uptake mechanisms, as well as the resulting cellular responses like toxicity. These interactions are highly dependent on both nanoparticle properties and the cellular microenvironment. ML approaches demonstrate strong capabilities in forecasting cellular association and uptake, and in identifying the influencing factors. For instance, a recent study utilized large-scale parallel screening and machine learning to systematically identify material properties and intrinsic cellular features (such as gene expression) that influence nanoparticle cellular uptake, constructing a genome-nanoparticle interaction network and identifying genetic biomarkers like SLC46A3^130^. This work effectively demonstrates how AI can leverage complex cellular data to predict nanoparticle absorption and pinpoint key influencing factors.

Crucially, AI is proving powerful in predicting nanoparticle cytotoxicity, a vital outcome of these cellular interactions and a key concern for therapeutic development. Based on experimental data from cell-based assays, ML models offer various capabilities for toxicity prediction. These include quantitative predictions, such as gradient boosting regression models demonstrating high accuracy in forecasting the viability of nanoparticle-treated cell lines^131^, and models providing quantitative cytotoxicity predictions across varying concentrations for inorganic nanomaterials^132^. Importantly, AI models also contribute to understanding the factors driving toxicity. By analyzing experimental data, they can identify key influencing attributes like size, surface properties, and experimental conditions, thereby highlighting the role of components such as nano-corona complexes in toxicity determination^133^. Leveraging these predictive and analytical capabilities, AI, often combined with techniques like genetic algorithms, enables high-throughput in silico screening. This allows for the rapid identification of selectively cytotoxic nanoparticles against specific cell lines^132^, significantly accelerating the search for targeted therapeutic candidates.

Understanding these intricate biological interactions across molecular and cellular scales through AI-driven prediction provides valuable insights into the fundamental mechanisms governing nanoparticle behavior, thereby guiding the rational design of nanomedicines with desired targeting and reduced side effects.

#### 4.2.3 Addressing key challenges in AI-driven theranostic design

However, despite the significant advancements and ongoing improvements in AI models for rational design and interaction prediction (as discussed in Section 4.2), building highly accurate predictive models with consistently good generalization ability across diverse chemical spaces and targets still faces significant challenges.

Firstly, a primary challenge stems from the availability and quality of training data. AI models are heavily reliant on large, high-quality datasets, yet data remains scarce, costly to acquire, and limited by privacy concerns and restricted sharing, especially for rare diseases or novel targets^134^. Available datasets often suffer from biases, errors, missing information, and inconsistent experimental results, further reducing AI reliability. This is particularly problematic for predicting novel structures or interactions with unknown targets, leading to poor performance^134^. As highlighted by some studies, this limitation is partly due to models sometimes tending to learn non-universal patterns or "shortcuts" from training data (for example, relying on the network topology of protein-ligand bipartite graphs rather than intrinsic molecular features)^117^, ^129^, which fundamentally limits their predictive capacity for truly novel compounds. Furthermore, a general lack of representation of 'negative data' (e.g., unsuccessful experiments) in literature hinders a complete understanding^134^.

Secondly, the interpretability and explainability of complex AI models remain a major hurdle. The "black box" nature of deep learning models makes it difficult to understand *why* a particular prediction is made, which is crucial for gaining biological insights, building trust among researchers and clinicians, and meeting regulatory requirements^134^.

Thirdly, computational intensity poses another severe challenge. While AI assists in pre-filtering candidates, traditional simulations like molecular docking still face huge bottlenecks when exploring ultra-large chemical spaces. Furthermore, training complex AI models requires substantial computational resources, creating barriers particularly for smaller research teams^134^.

Finally, balancing multiple objectives in the rational design phase (e.g., optimizing simultaneously for activity, selectivity, ADMET, and synthesizability) and developing robust, standardized evaluation processes for models across these diverse criteria remains complex^134^.

In summary, while AI models have shown immense potential in predicting nanomedicine properties and interactions, effectively overcoming these multifaceted challenges is paramount. These hurdles necessitate innovative strategies that integrate computational predictions with experimental validation, forming a dynamic and iterative workflow.

### 4.3 Creating a closed-loop theranostic system for iterative optimization

Capitalizing on the enhanced predictive capability of AI, a natural synergy is formed with the screening and modeling platforms introduced in Sections 2 and 3. This potent combination is what closes the theranostic loop: AI processes the 'diagnostic' data from the screening platforms to build predictive models, and then leverages these models to guide the rational design of the 'therapeutic' agent. This directly enables the building of a dynamic, self-optimizing R&D system. As highlighted in relevant reviews^117^, closely integrating computational tools with experimental validation is considered crucial for driving the transformation of drug discovery processes, aiming to overcome the bottlenecks of traditional linear workflows and form a "virtuous cycle" where experimental data continuously refines computational models, and in turn, models guide experiments **(Table 4)**.

The core workflow of this AI-driven closed-loop system was envisioned as follows:

**AI Design and Prediction Phase:** In this initial phase, AI models, leveraging prior knowledge and learned patterns (including those from biological atlases^118^, ^135^, ^136^ and previous experimental cycles), predicted the potential performance of a series of virtually designed or initially screened nanomedicines or their components for brain targeting. Advanced generative models could even explore novel chemical spaces and propose innovative nanomedicine design solutions with predicted properties (as discussed in Section 4.2.1). These *in silico* predictions aimed to rapidly narrow down the potential design space, prioritizing candidates with the highest predicted potential based on properties like BBB permeability and target interaction (as discussed in Section 4.2.2).

**Automated Synthesis Phase:** Subsequently, based on AI's predictions and rational design suggestions, the (automated) nanomedicine synthesis phase was initiated. This phase utilized automated synthesis platforms (such as robotic synthesisers) for high-throughput, high-precision synthesis of selected nanomedicine candidates, ensuring batch-to-batch consistency and reproducibility^137^, ^138^. High-throughput synthesis was essential to generate the diverse set of candidates needed for the subsequent experimental evaluation.

**Experimental Testing (HTS/HCS) and AI-Integrated Optimization Phase:** Next, these newly synthesized nanomedicines underwent systematic evaluation using high-fidelity experimental platforms. This involved applying HTS/HCS to large-scale brain organoid models or BOoC platforms and employing methods like high-content imaging, multi-omics analysis (including single-cell omics), and functional assays to comprehensively collect detailed experimental data on the interactions and effects of the nanomedicines within the complex brain organoid microenvironment. This experimental phase actively leveraged AI-integrated High-Throughput Experimentation (HTE) strategies not only for data acquisition but also for accelerated optimization and understanding structure-activity relationships (SARs) within the complex nanomedicine design space. Computation was integrated with HTE to guide the experimental design itself and analyze the resulting data for optimization. For instance, a landmark study demonstrated how the integration of machine learning with high-throughput experimentation enabled the rapid design and discovery of novel self-assembling nanoparticle formulations from a vast chemical space^139^. By computationally predicting promising drug-excipient combinations and then rapidly validating nanoparticle formation and properties using high-throughput methods, they effectively explored millions of potential formulations. Similarly, other work applied this integrated approach (HTS synthesis/screening combined with machine learning) to explore the design space of complex nanostructures like spherical nucleic acids (SNAs), revealing structure-activity relationships and identifying key design parameters influencing biological activity with significantly fewer tests than traditional methods^140^. These specific examples illustrate *how* the deep integration of AI and HTE within the experimental cycle overcomes complexity and inefficiency by allowing for data-driven exploration and optimization of the vast design space and facilitating the discovery of underlying design rules.

**Data Feedback and AI Model Learning/Update Phase:** The massive, high-quality, high-dimensional experimental data generated by the HTS/HCS platform, incorporating these AI-integrated HTE strategies, was then collected, processed, and standardized as HTS data feedback, which was subsequently "fed" to the AI system. The experimental data from advanced *in vitro* models like brain organoids or organoids-on-a-chip provided more biologically relevant data for training AI models (e.g., models for predicting ADMET/PK properties, cellular uptake, efficacy, or SARs), thereby significantly improving the quality and predictive power of the models compared to using less relevant data. Finally, in the AI model learning and update phase, AI models utilized this new stream of data for iterative optimization. They continuously updated their internal parameters and improved their predictive accuracy and understanding of complex biological laws governing nanomedicine behavior in the brain context. If the model's predictions for a tested nanomedicine did not match the experimental results, the AI model performed self-adjustment and refinement using the discrepancy as a learning signal. If the predictions matched, the model's confidence was further strengthened.

The AI model, after learning and updating, was then used to guide the next round of nanomedicine design, prediction, and screening, potentially exploring modifications to the previous candidates or proposing entirely new designs based on the improved SAR understanding. This process formed a continuously iterative, spiraling upward closed-loop, where each cycle built upon the knowledge gained in the previous one, leading to progressively better designs and more accurate predictions, thereby accelerating the path to identifying promising candidates.

## 5. Charting the path toward personalized nanotheranostics

The preceding sections detailed the complexity of brain diseases and the challenges of brain-targeted nanomedicine delivery. We explored the significant potential of three key areas: high-fidelity brain organoid models; HTS/HCS technologies which generating rich, multi-dimensional data; and AI that serves as a core engine for extracting insights and guiding rational design. These technologies, developed independently or synergistically, together lay a solid foundation for the future development of brain-targeted nanomedicine, heralding a new era of therapy that is more precise, efficient, and even tailored to individuals. Deeply integrating these cutting-edge technologies is expected to overcome current bottlenecks in brain nanomedicine R&D, significantly improving clinical translation efficiency and treatment success rate **(Table 5)**.

### 5.1 Advancing the precision of nanomedicine through a theranostic approach

Looking ahead, AI-driven intelligent design and screening engines will be deeply coupled with high-fidelity brain organoid models, greatly enhancing the precision of predicting the behavior and delivery of brain-targeted nanomedicines in the complex brain environment. The high complexity of brain structure, cellular heterogeneity across different brain regions, disease heterogeneity, and the strict limitations of the Blood-Brain Barrier (BBB) require nanomedicines to precisely cross the BBB, reach specific brain regions, and specifically target diseased cells or subcellular structures while avoiding impact on healthy brain tissue. Achieving this high level of precision particularly relies on gaining deep understanding of the molecular characteristics of various cell types and subtypes making up brain tissue, as well as their features in different brain regions and physiological/pathological states. Recently, large-scale single-cell genomics research has made breakthroughs, constructing a comprehensive brain cell atlas covering major human brain regions, developmental stages, and disease states^118^, as well as high-resolution atlases specifically for the human brain vasculature^135^, ^136^. These atlases revealed with unprecedented detail the wide diversity of brain cell types and subtypes, region-specific differences across brain regions, and disease-specific molecular alterations, providing a key reference for precisely defining the cell types and subtypes that need to be targeted and understanding their molecular basis. Utilizing the molecular details revealed by these human brain cell atlases, combined with AI predictive models, researchers can precisely predict their BBB permeability, brain distribution patterns, and interactions with and uptake efficiency by specific brain cell types like neurons, glial cells, and vascular endothelial cells based on nanomedicine structural parameters (e.g., size, surface chemical modification, charge), drug loading properties, and characteristics of the simulated brain microenvironment. Further combining diverse brain organoid models with high-content screening to validate these predictions will enable the design of brain-targeted nanomedicines to truly achieve precise control from macroscopic distribution to microscopic cell/subcellular localization, maximizing efficacy and minimizing off-target effects.

### 5.2 Streamlining the R&D pipeline for faster theranostic development

Besides enhancing the precision of drug delivery, the application of AI is driving a significant transformation in the entire drug development process, significantly improving efficiency and effectiveness in key areas including virtual screening, ADMET prediction, and synthesis planning^141^. In the field of brain nanomedicines, which is characterized by a long R&D cycle and high costs, the AI-driven closed-loop learning and iterative optimization workflow discussed earlier is the core approach to achieving efficiency breakthroughs. Unlike the traditional lengthy linear R&D model, the AI-enabled workflow can rapidly perform computational prediction and rational design, significantly narrowing down the range of candidates before entering the experimental stage and prioritizing nanomedicine formulations and compositions with high potential for success. This rapid computation-experiment iterative cycle significantly reduces unproductive experiments, greatly shortening the cycle time from lead compounds to preclinical candidates, lowering R&D costs, and thereby accelerating the discovery and translation of brain-targeted nanomedicines.

The potential of AI to transform and accelerate the entire drug discovery and development pipeline has been clearly demonstrated by successful cases, even outside the specific realm of nanomedicine. For example, a compelling recent case illustrated how an AI platform was used to identify TNIK as a potential therapeutic target for fibrosis^141^, ^142^. Subsequently, through an AI-assisted structure-based design workflow, a small molecule inhibitor targeting TNIK was rationally designed and optimized. This AI-driven design process considered molecular structural features, predicted binding modes with the target, and predicted ADMET/PK properties. The lead compound identified through this computationally guided process was effectively validated in subsequent *in vitro* and *in vivo* experiments, demonstrating favorable efficacy and safety profiles, and was ultimately successfully advanced to clinical trials. This demonstrably successful AI-driven drug development workflow, while applied to a small molecule drug, highly aligned with the principles of AI-driven design proposed in this review for brain-targeted nanomedicines, leveraging computational guidance for rational design and accelerated progression through the pipeline. This case validated AI's potential in guiding drug discovery and optimization across the entire pipeline, a principle directly applicable to accelerating the development of brain nanomedicines within the proposed new paradigm.

### 5.3 Realizing personalized theranostics for individual patient care

**Figure 5:**
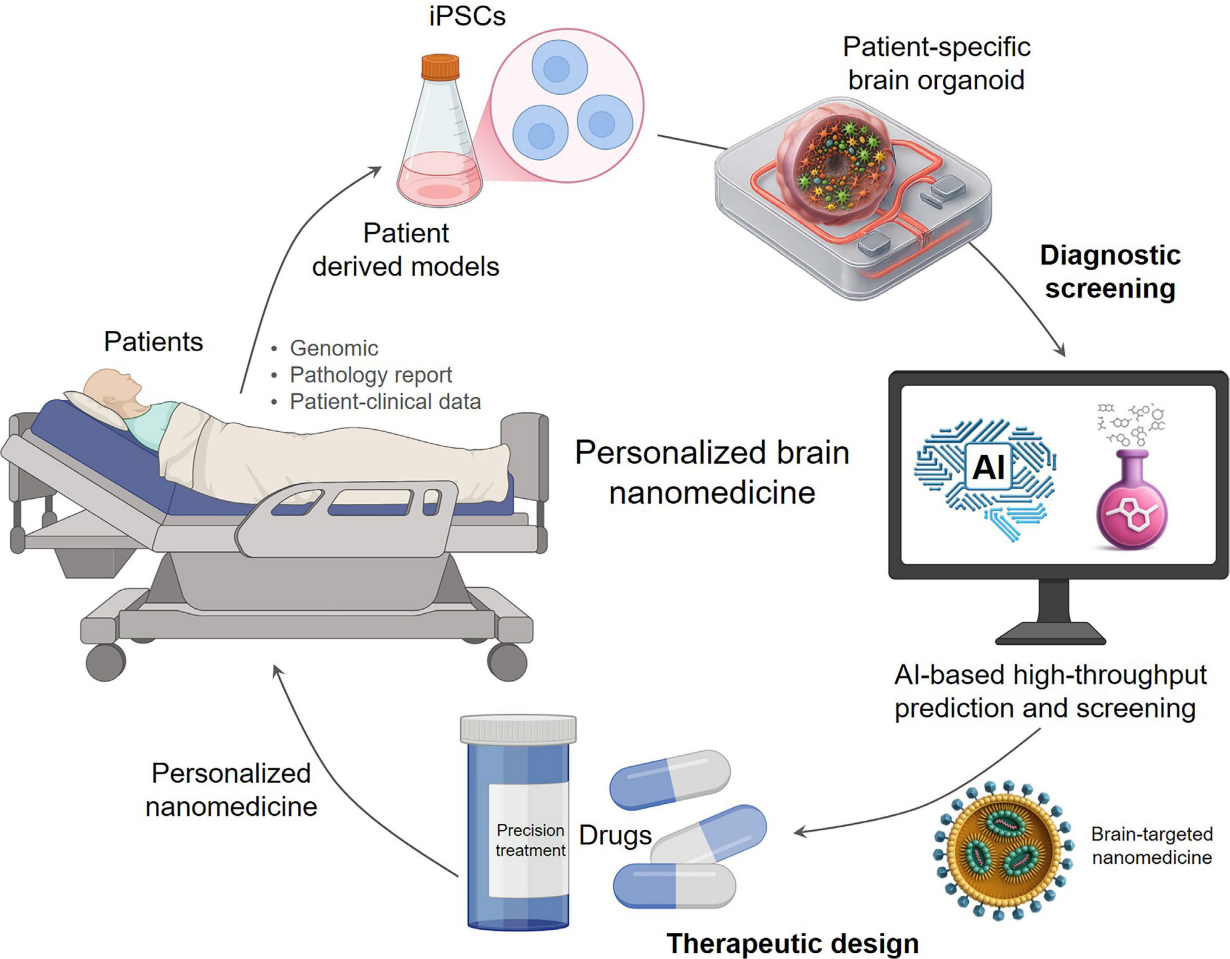
** The proposed workflow for personalized brain theranostics.** This figure illustrates the future of individualized patient care, enabled by a closed-loop theranostic workflow. The cycle begins with an individual patient, from whom clinical and biological data (e.g., genomic, pathology reports) are collected. This information is used to engineer a 'theranostic digital twin'-a patient-specific brain organoid model that recapitulates the individual's disease. This high-fidelity model is then subjected to an AI-driven high-throughput screening platform, which performs two key tasks: (1) 'diagnosing' the organoid's response to a library of nanomedicines and (2) 'guiding' the rational design of an optimal, personalized nanotherapeutic. Finally, the resulting tailor-made nanomedicine is administered to the patient, completing the patient-centric theranostic cycle from diagnosis to precision treatment.

Ultimately, combining AI-driven intelligent design, efficient screening, and high-fidelity models is expected to achieve precise treatment for individual patients with brain diseases **(Figure 5)**. Brain diseases commonly exhibit significant inter-patient variability, reflected in genetic background, pathological features, disease progression, and treatment response, making traditional "one-size-fits-all" treatment approaches difficult to be effective. Achieving individualized medicine requires developing treatment plans based on the patient's unique biological characteristics. By combining patient genomic, clinical imaging, and liquid biopsy data, as well as data from patient-specific brain organoid models^143^, ^144^, AI models can deeply learn and model patient-level disease features and response patterns to different nanomedicines. This approach effectively transforms each patient-derived organoid into a 'theranostic digital twin.' This powerful *in vitro* proxy allows clinicians to computationally screen and rationally design the optimal nanomedicine for that specific individual before administration, seamlessly integrating personalized diagnosis and treatment. Recently, researchers developed individualized patient tumor organoid (IPTO) models, constructed by co-culturing patient tumor tissue with brain organoids. These models faithfully preserved the human brain tumor microenvironment and cellular heterogeneity and were proven capable of accurately predicting patient clinical response to therapies like temozolomide (TMZ) in prospective clinical studies and revealing potential resistance mechanisms^143^. This breakthrough achievement validated the feasibility of using high-fidelity individualized models to predict real patient treatment response. In the future, combined with AI's deep analysis, pattern learning, and prediction of data generated from these individualized models, AI will be able to provide decision support for clinicians, assisting in selecting the most suitable brain-targeted nanomedicine formulation and treatment plan for specific patients, thereby truly achieving precise treatment for individualized brain diseases, maximizing efficacy and minimizing side effects.

## 6. Conclusion

Developing effective therapies for CNS diseases remains a formidable challenge, primarily due to the brain's biological complexity, the stringent BBB, and the limitations of traditional R&D models. This review has presented an integrated paradigm leveraging cutting-edge technologies to overcome these hurdles. The core of this approach lies in the synergy between high-fidelity brain organoid models (including organoid-on-a-chip platforms), HTS/HCS, and AI.

This powerful technological system enables a dynamic, self-optimizing closed-loop R&D workflow. High-fidelity models generate biologically relevant, high-dimensional data on nanomedicine interactions within a complex brain-like environment. AI serves as the intelligent engine, processing this data to build predictive models, inform rational nanomedicine design, and guide rapid experimental validation via HTS/HCS.

The adoption of this integrated paradigm promises to significantly accelerate brain-targeted nanomedicine discovery and improve its precision. It allows for more accurate prediction of nanomedicine behavior, including BBB permeation, tissue distribution, and cell-specific targeting. By intelligently navigating the vast design space and iteratively refining candidates based on experimental feedback, the R&D cycle can be substantially shortened, reducing costs and increasing success rates. Furthermore, the potential to integrate patient-specific data and models offers a transformative pathway towards truly individualized brain disease treatment.

While the convergence of these fields marks a pivotal shift, the path toward realizing this fully integrated paradigm is not without significant hurdles that require concerted effort to overcome. Key challenges remain in standardizing the highly complex and multi-modal experimental data generated from diverse organoid platforms to ensure quality and enable robust cross-platform validation. A potential solution lies in collaborative, community-wide efforts to establish standardized data formats and build open-access, federated databases.

Concurrently, enhancing AI model generalization and interpretability is paramount; overcoming the "black box" nature of deep learning is critical for building trust and deriving actionable biological insights. The development and application of explainable AI (XAI) will be essential in this regard^145^. Perhaps most critically, we must ensure the clinical relevance of this entire workflow by rigorously validating that the insights gleaned from these in vitro systems possess true predictive power for outcomes in human patients. This will require systematic studies correlating organoid-derived biomarkers and therapeutic responses with real-world clinical data. Despite these challenges, this AI-driven, integrated approach provides a robust framework and unprecedented tools for developing precise, efficient, and personalized brain-targeted nanomedicines, holding great promise for addressing devastating neurological conditions in the future.

## Figures and Tables

**Table 1:** Key human brain organoid technologies for theranostic modeling

Organoid type	Source & method	Key features & advantages	Primary applications	Pathologies modeled	Ref
Whole-Brain	PSCs via undirected 3D self-organization	Models global brain development & early inter-regional interactions	Studying neurodevelopment; investigating inter-regional signaling	Microcephaly, viral encephalopathies	38
Region-Specific	PSCs via directed differentiation using region-specific signaling	High regional specificity and structural precision	Region-specific disease modeling; targeted drug testing	Neurodegenerative diseases (e.g., Alzheimer's)	48
Choroid Plexus (ChP)	PSCs via ChP-specific induction	Forms a functional blood-CSF barrier (B-CSF-B) *in vitro*	Screening drug CNS permeability; studying barrier function	CNS barrier disorders	55
Genetically Engineered	iPSCs + Gene Editing (e.g., CRISPR-Cas9)	High-fidelity modeling of genetic diseases; enables isogenic controls	Personalized medicine; anti-cancer drug screening	Pediatric brain tumors, hereditary syndromes	69
High-Reproducibility	Methodological enhancement (e.g., "Hi-Q" protocol)	Improved reproducibility, scalability, and diversity for HTS	High-throughput screening (HTS) platforms	Glioma invasion, microcephaly	54
Explant-Derived	Direct culture of fetal brain tissue	Preserves native tissue architecture and enables direct disease gene introduction	Modeling tumorigenesis	Brain tumors	146

**Table 2:** Summary of Brain-Organoid-on-a-Chip (BOoC) technologies

Platform	Key components	Method	Core advantage	Primary application	Key research goal	Ref
Basic BOoC	Brain Organoid + Microfluidic Chip	Integrating organoids into a perfused microfluidic chip.	Overcomes static culture limitations (e.g., nutrient diffusion, necrotic cores); provides a dynamic, controlled microenvironment.	Enhanced, long-term brain modeling.	Improving model fidelity for studying neuropathology.	93
BOoC-BBB Model	BOoC + BBB-specific cells (endothelial cells, pericytes, astrocytes).	Co-culture of organoids with key BBB cell types on a single chip.	Recreates a functional BBB *in vitro*; enables dynamic study of nanoparticle transcytosis.	Testing drug and nanoparticle delivery across the BBB.	Modeling neurovascular unit (NVU) dysfunction; studying transport mechanisms.	94
Multi-Organon-a-Chip	BOoC fluidically linked with other organ modules (e.g., liver, kidney).	Creating interconnected, multi-organ systems on a chip.	Simulates systemic drug effects (ADMET) and inter-organ crosstalk, providing greater physiological relevance.	Pharmacokinetic (PK/PD) modeling; systemic toxicology screening.	Understanding whole-body response and off-target effects of nanomedicines.	95

**Table 3:** Achieving scale and depth in organoid screening with HTS/HCS.

Technology	Core function	Challenge overcome	Key applications	Ref
High-Throughput Screening (HTS)	Enables scale and automation to generate, culture, and process thousands of organoids in a standardized manner.	Throughput Bottleneck: Overcomes the low-volume, manual limitations of traditional organoid research.	Large-scale chemical/drug screening; high-throughput evaluation of nanomedicines (e.g., BBB permeability, toxicity).	103
High-Content Screening (HCS)	Enables deep and quantitative data acquisition by automatically capturing complex, multi-dimensional phenotypic data.	Lack of Data Depth: Moves beyond simple viability readouts to capture rich, multi-parameter biological information.	Multi-modal phenotyping (imaging, electrophysiology); quantitative analysis of complex disease pathologies (e.g., Aβ/p-tau).	105
HTS/HCS + Single-Cell Omics	Reaches molecular-level resolution by revealing cellular responses and mechanisms for individual cells.	Cellular Heterogeneity: Dissects cell-type-specific responses that are masked in bulk analysis.	Drug target identification and validation (e.g., CRISPR screens); deciphering gene regulatory networks.	106

**Table 4:** AI's role in orchestrating the brain-targeted nanomedicine R&D workflow.

AI-driven phase	Core function & purpose	Key AI technologies	Ref
Data Processing & Feature Extraction	Process massive, high-dimensional HTS/HCS data (images, omics) to extract meaningful biological features for model training.	Convolutional Neural Networks (CNNs) for image analysis; algorithms for segmenting and quantifying cellular features.	113
Predictive Modeling & Rational Design	Build predictive models for nanomedicine properties (e.g., BBB permeability, toxicity) and guide the *de novo* or rational design of new candidates.	QSNAP for self-assembly prediction; AI-enhanced Virtual Screening (VLS); Modular Synthon-Based Design.	119
Closed-Loop Theranostic Cycle	Create a self-optimizing "Design-Build-Test-Learn" cycle by integrating AI-driven design, automated synthesis, and HTS/HCS testing.	Generative AI for *de novo* design; ML-integrated High-Throughput Experimentation (HTE); robotic synthesis platforms.	139
Overarching Challenges	- Data Scarcity & Quality; Model Interpretability (the "black box" problem); High Computational Cost. -		134

**Table 5:** The path to precise and personalized brain nanomedicine.

Future application	Key enabling technologies	Landmark example discussed	Theranostic goal	Ref
Precision Design & Delivery	AI Models + High-Resolution Brain Cell Atlases + High-Fidelity Organoids	Comprehensive Brain Atlases: Single-cell atlases of human brain and vasculature provide molecular maps for targeting.	Achieve nanomedicine delivery with cell-type and subcellular precision, maximizing efficacy and minimizing off-target effects.	118
Accelerated R&D Pipeline	AI-driven Closed-Loop "Design-Validate" Platforms	AI-Discovered TNIK Inhibitor: An AI platform identified a novel target and guided inhibitor design, which rapidly advanced to clinical trials.	Significantly shorten discovery-to-clinic timelines, reduce costs, and increase the success rate of brain-targeted nanomedicines.	141
Individualized Theranostics	Patient-Specific Organoid Models ("Digital Twins") + AI-driven Analysis	Individualized Patient Tumor Organoids (IPTOs): Patient-derived models accurately predicted clinical responses to therapy (TMZ).	Enable true personalized medicine by selecting or designing the optimal nanomedicine for each individual patient's disease.	143
